# Genetic diversity and population structure of sheep (*Ovis aries*) in Sichuan, China

**DOI:** 10.1371/journal.pone.0257974

**Published:** 2021-09-28

**Authors:** Mingliang Zhou, Gaofu Wang, Minghua Chen, Qian Pang, Shihai Jiang, Jie Zeng, Dan Du, Pinggui Yang, Weisheng Wu, Hongwen Zhao

**Affiliations:** 1 Sichuan Academy of Grassland Sciences, Chengdu, Sichuan, China; 2 Chongqing Academy of Animal Sciences, Chongqing, China; Sant Baba Bhag Singh University, INDIA

## Abstract

Sichuan, China, has abundant genetic resources of sheep (*Ovis aries*). However, their genetic diversity and population structure have been less studied, especially at the genome-wide level. In the present study, we employed the specific-locus amplified fragment sequencing for identifying genome-wide single nucleotide polymorphisms (SNPs) among five breeds of sheep distributed in Sichuan, including three local pure breeds, one composite breed, and one exotic breed of White Suffolk. From 494 million clean paired-end reads, we obtained a total of 327,845 high-quality SNPs that were evenly distributed among all 27 chromosomes, with a transition/transversion ratio of 2.56. Based on this SNP panel, we found that the overall nucleotide diversity was 0.2284 for all five breeds, with the highest and lowest diversity observed in Mage sheep (0.2125) and Butuo Black (0.1963) sheep, respectively. Both Wright’s fixation index and Identity-by-State distance revealed that all individuals of Liangshan Semifine-wool, White Suffolk, and Butuo Black sheep were respectively clustered together, and the breeds could be separated from each other, whereas Jialuo and Mage sheep had the closest genetic relationship and could not be distinguished from each other. In conclusion, we provide a reference panel of genome-wide and high-quality SNPs in five sheep breeds in Sichuan, by which their genetic diversity and population structures were investigated.

## Introduction

Modern sheep (*Ovis aries*) were domesticated in the Fertile Crescent region of the Near East approximately 8,000–9,000 years ago [1]. The comprehensive analysis by genotyping 50K single nucleotide polymorphism in 2,819 animals from 74 worldwide sheep breeds revealed that they had maintained a relatively high genetic diversity in comparison with other main farm animals [2]. China has relatively abundant sheep genetic resources composed of at least15 officially recognized breeds and many local populations [3]. Although Chinese sheep breeds may be classified geographically and morphologically classified into three groups, namely Mongolian, Kazakh, and Tibetan, molecular data from 10 representative indigenous sheep breeds showed that they might actually be divided into two genetic groups [4], that is, the thin (Tibetan group) and the fat (Mongolian and Kazakh group) types. Overall, a large number of sheep breeds are widely distributed in a diverse range of environments with considerable genetic and morphological variation.

According to our previous investigation, there are at least eight local breeds of sheep in Sichuan province, China, and all of them have particular morphological characteristics and geographical distribution. For example, Butuo Black sheep has are geographically distributed in Butuo county of Liangshan Yi Autonomous Prefecture of southwestern Sichuan; the average adult male and female body weights are 53.51 ± 8.44 kg and 39.08 ± 7.63kg, respectively [5]. Mage is a recently recognized and small dual-purpose (wool/meat) sheep breed in Derong county of Ganzi Tibetan Autonomous Prefecture. In addition to these local breeds, many exotic sheep breeds, such White Suffolk, have been raised because of their high production performance. However, to the best of our knowledge, there has been no previous study that systematically investigated the genetic diversity and population structures of these local sheep breeds in Sichuan.

Given the rapid advances in high-throughput DNA sequencing technologies [6], it has become EASIER and more cost-effective to identify a large number of genome-wide SNPs that could be used to study genetic diversity and population structures. Among these technologies, specific-locus amplified fragment sequencing (SLAF-seq) could produce the evenly distributed SNPs through whole genome at a very low cost [7]. In the present study, we also employed the SLAF-seq to investigate the genetic diversity and population structures of five sheep breeds distributed in Sichuan, including three local pure populations.

## Materials and methods

### Ethics statement

Sample collection and study purposes were approved by the Research Committee of Sichuan Academy of Grassland Sciences, Sichuan, China.

### Breeds and animals

**Fig 1:**
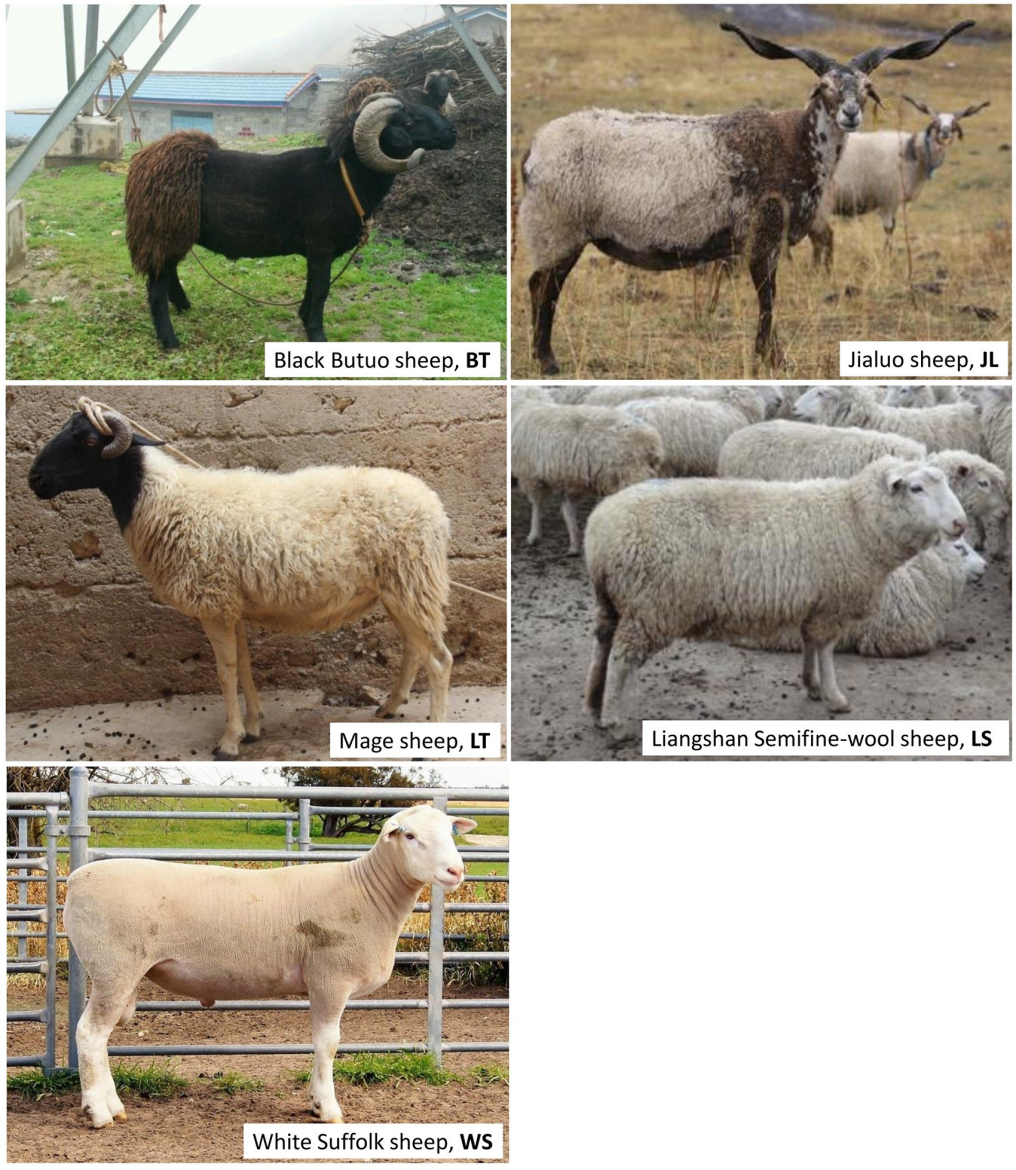
The five sheep breeds in Sichuan province.

We collected a total of 69 blood samples from five different sheep breeds in the Sichuan province (Fig 1), consisting of (1) three local pure breeds, namely Butuo Black (BT, N = 9), Jialuo (JL, N = 8), and Mage (LT, N = 19), (2) a composite Liangshan Semifine-wool sheep (LS, N = 19), and (3) the recently introduced White Suffolk (WS, N = 14). To ensure good representation of diversity, we verified that the selected animals within each breed had no genetic relationships with those within other breeds for three generations, according to their pedigree records. Morphological characteristics were carefully checked to avoid possible hybrid relationships with other breeds.

### SLAF-seq

Genomic DNA was extracted from the blood samples using Axy-Prep Genomic DNA Miniprep Kit (Axygen Bioscience, Tewksbury, MA, USA) and evaluated for quality using agarose gel electrophoresis. According to the recommended pipeline, ~1 μg genomic DNA was incubated at 37°C with *EcoR*I (New England Biolabs [NEB], Ipswich, MA, USA), T4 DNA ligase (NEB), ATP (NEB), and adapter. After heat-inactivating ligation reactions at 65°C, the samples were digested with *Alu*I at 37°C. Polymerase chain reaction was performed using diluted restriction-ligation samples, dNTP, Taq DNA polymerase (NEB), and *EcoR*I-primer containing barcode1. The purified samples were subjected to the fragment selection for ~450 bp using a Gel Extraction Kit (Qiagen, Hilden, Germany). These fragment products were then subjected to polymerase chain reaction using Phusion Master Mix (NEB) and Solexa Amplification primer mix to add barcode2. The sequencing libraries were obtained using the Illumina HiSeq platform, and 100 bp paired-end reads were generated (Biomarker, Beijing, China).

### SNP calling

For the raw sequencing reads, the Q_phred_ value-based error rate and GC content were analyzed. Using the fastp tool [8], low-quality reads were filtered out if they belonged to any one of the following three types: (1) reads containing adaptor sequences, (2) reads containing unambiguous bases of N more than 10% of the total length, and (3) reads containing low-quality bases (Q <5) more than 50% of the total length. If any member of a paired read was marked as low quality, both pairs were discarded. After these steps, we obtained clean reads for subsequent analyses. Reads were aligned to the reference genome of sheep (Oar_rambouillet_v1.0 retrieved from NCBI) using BWA-MEM algorithm and default parameters in the BWA tool [9]. Picard tools (v1.134; http://broadinstitute.github.io/picard/) were employed to remove duplicate reads and sort the Sequence Alignment/Map files. Subsequently, SNPs were called and genotyped using GATK software (v3.7) according to GATK Best Practices recommendations [10–12].

### Data analysis

All clean SNPs were individually annotated using the genome annotation file in GTF format and analysis of variance tool [13]. We analyzed the read coverage, chromosomal distribution, nucleotide diversity (*π*), observed heterozygosity (*H*_*o*_), and expected heterozygosity (*H*_*e*_), Hardy–Weinberg equilibrium, and pairwise Identity-by-State (IBS) distance using the VCF tools and Plink (Danecek, 2011). The polymorphism information content (PIC) and Wright’s fixation index (F_ST_) of the breeds were computed using the PopSc toolkit [14].

## Results

**Fig 2:**
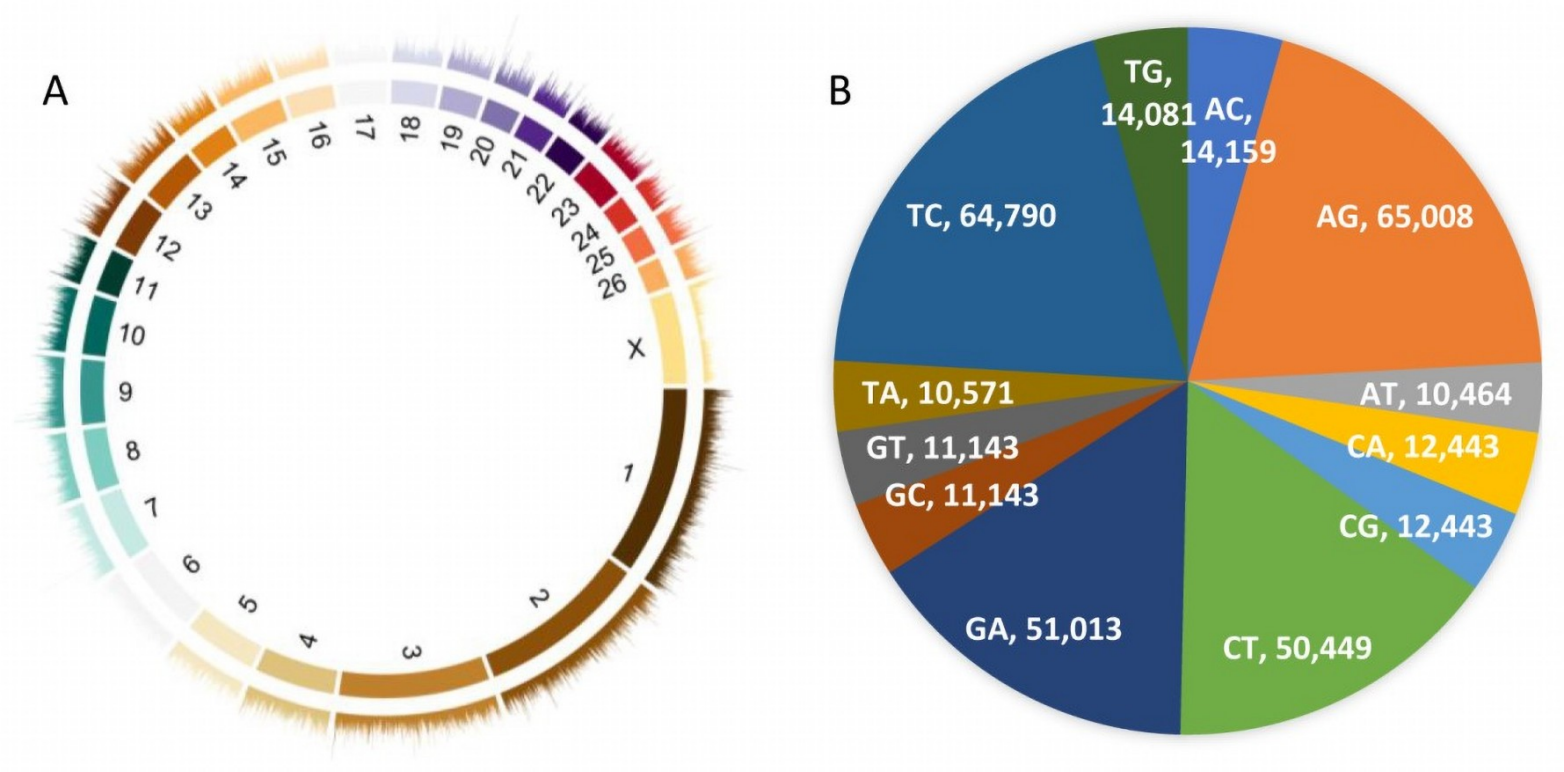
Distribution density across 27 chromosomes (A) and substitution types (B) of clean single nucleotide polymorphisms (SNPs) in this study.

**Fig 3:**
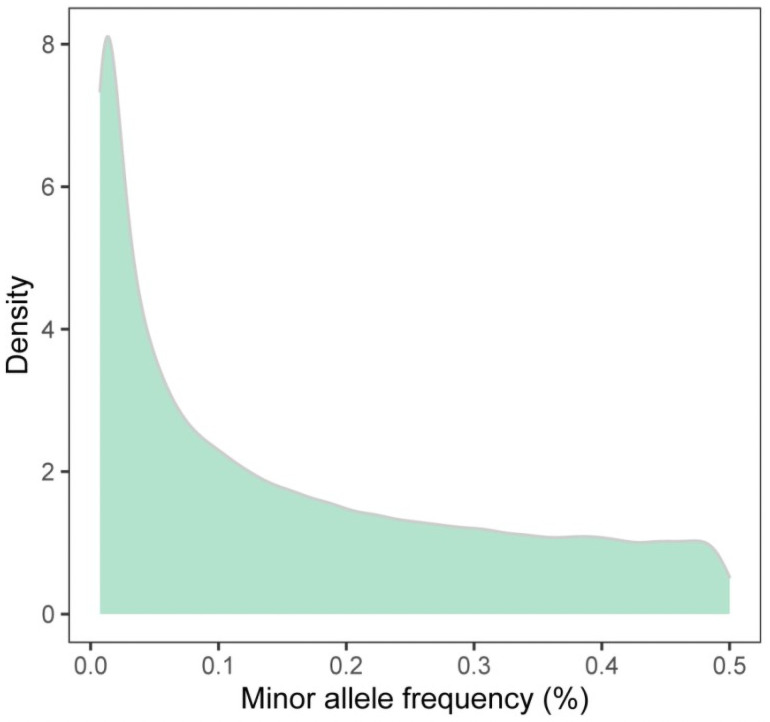
Distribution density of minor allele frequencies.

We obtained 507 million raw paired-end reads in total and 494 million clean reads after quality filtering (S1 Table). The average rates of alignment and unique alignment of reads to the reference genome sequence was 96.53% and 93.07%, respectively. After quality filtering, a total of 327,845 SNPs were detected among all the 69 samples, and all of them had an average read coverage of 23.75X. According to the positional annotation, all these SNPs were located within intergenic regions (9.068%), exons (0.826%), introns (36.229%), ncRNA transcript regions (36.560%), and 5′/3′ untranslated regions (UTRs; 0.771%). With respect to the whole genome, all SNPs were evenly distributed among the 27 chromosomes, with the highest and lowest counts in chromosomes 1 (N = 34,523) and 24 (N = 5,819), respectively (Fig 2A). The overall transition/transversion ratio was 2.559 with 7,303,562 transitions and 2,854,530 transversions detected (Fig 2B). The frequency distribution of minor alleles among all the clean SNPs is shown in Fig 3.

Based on all the clean SNPs, we calculated the genetic diversity for individual and pooled breeds (Table 1). The overall nucleotide diversity was 0.2284 for all the five breeds, with the highest and lowest diversity observed in Mage (0.2125) and Butuo Black (0.1963) sheep, respectively. All the five breeds had *H*_*o*_ lower than *H*_*e*_, and the largest and smallest differences were observed in Jialuo (0.1507 vs. 0.2212) and Liangshan Semifine-wool (0.1918 vs. 0.2132) sheep, respectively. The PIC ranged from 0.1395 in Jialuo sheep to 0.2492 in Liangshan Semifine-wool sheep, with the average value of 0.2217 among the five breeds.

**Table 1:** Genetic diversity among the five sheep breeds.

Breed	Nucleotide diversity	Observed heterozygosity	Expected heterozygosity	Polymorphism information content
**BT**	0.1963	0.1763	0.2090	0.1469
**JL**	02055	0.1507	0.2212	0.1395
**LT**	0.2125	0.1635	0.2195	0.2433
**LS**	0.2077	0.1918	0.2132	0.2492
**WS**	0.2047	0.1543	0.2131	0.1975
**All**	0.2284	0.1703	0.2302	0.2217

BT, Butuo Black sheep; JL, Jialuo sheep; LT, Mage sheep; LS, Liangshan Semifine-wool sheep; WS, White Suffolk.

**Fig 4:**
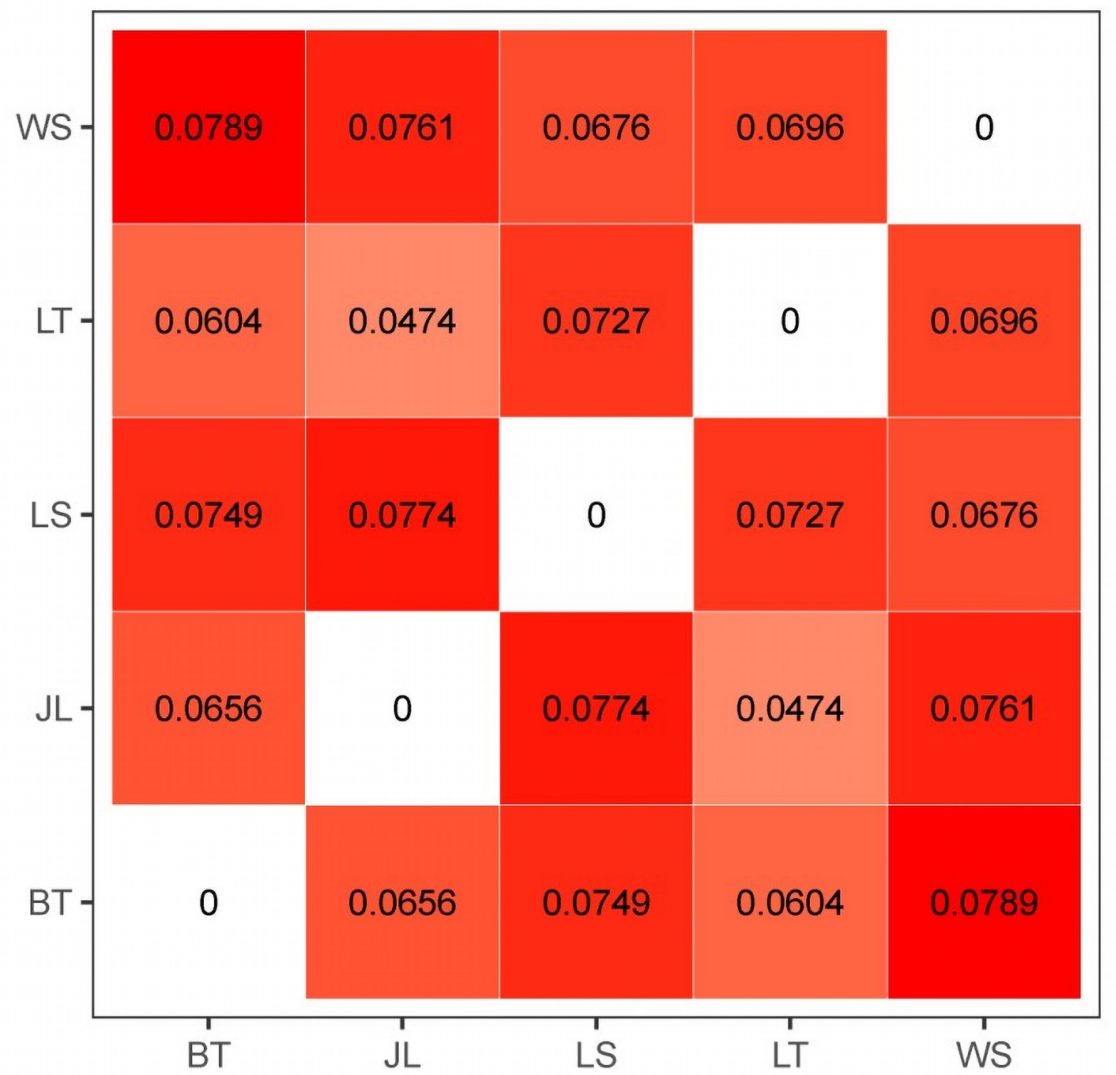
Pairwise wright’s F_ST_ values among the five breeds.

**Fig 5:**
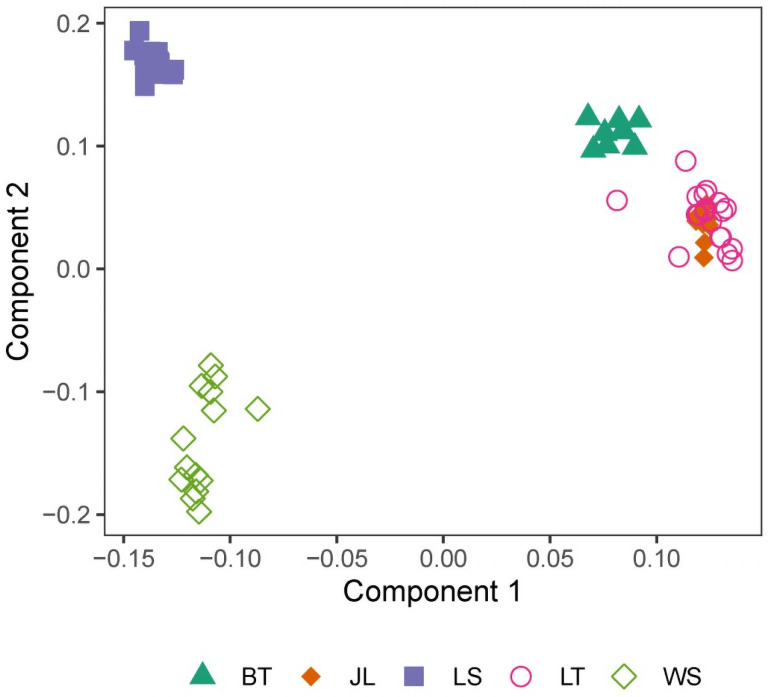
Sample clustering by IBS distance-based PCA plotting.

Among all the 69 samples, 122,816 SNPs were revealed to be in Hardy-Weinberg equilibrium, with *P*-value < 0.05. The pairwise F_ST_ values among the five breeds are shown in Fig 4. The most significant genetic divergence was observed between Butuo Black and White Suffolk sheep (F_ST_ = 0.0789), whereas the lowest F_ST_ was 0.0474 (Mage vs. Jialuo sheep). Among the three local pure breeds of Butuo Black, Jialuo, and Mage sheep, the pairwise genetic divergences slightly ranged from 0.0656 to 0.0774 in terms of F_ST_ value. PCA-based clustering was performed based on the pairwise IBS distances (Fig 5). It was clearly revealed that all individuals of Liangshan Semifine-wool, White Suffolk, and Butuo Black sheep were clustered together and obviously separated from each other. However, all individuals of Jialuo and Mage sheep were clustered together and could not be distinguished from each other.

## Discussion

Given the climate changes and increasing threats to intensively selected composite animal breeds, it has become urgent to effectively conserve local genetic resources of domestic animals. In particular, considerable efforts have been made in field to investigate novel and valuable local breeds of sheep in Sichuan, China [5]. This province has abundant genetic resources of sheep and other farm animals, mainly due to its highly variable environmental conditions [3]. Further, sheep breeds in Sichuan have unique morphological characteristics and geographic distribution. However, the genetic diversity and population structures have not been systemically investigated yet, especially at the genome-wide level. In this study, we successfully employed the high-throughput sequencing technology SLAF-seq and found a large number of evenly distributed genome-wide and high-quality SNPs in five sheep breeds, including three local pure breeds, one composite breed (Liangshan Semifine-wool sheep), and one recently introduced exotic breed (White Suffolk) [7]. Based on these molecular markers, the genetic diversity and population structures of the sheep breeds were evaluated. To the best of our knowledge, this is the first report on the genetic diversity of Sichuan sheep breeds based on genome-wide SNPs.

Wang and Wu (2018) used 12 microsatellite markers and investigated the genetic diversity of six sheep breeds in Sichuan [15], which revealed a PIC of 0.559~0.612 and *H*_*e*_ of 0.610~0.670. Using 30 microsatellite markers, we previously found that the genetic diversity of Butuo Black sheep was 0.6257 for PIC and 0.6269 for *H*_*o*_ [16]. In the present study, we observed that Butuo Black sheep had the lowest genetic diversity in comparison with the other four breeds, including the composite breed of Liangshan Semifine-wool sheep and the exotic breed of White Suffolk sheep. The results suggest relatively high genetic diversity of sheep breeds in Sichuan, which is consistent with a recent publication investigating the national sheep breeds in China [17]. We also observed that, in addition to local pure breeds, the recently introduced and composite sheep breeds in Sichuan have a relatively high genetic diversity; this would be favorable in reducing potential inbreeding depression.

Among the five sheep breeds included in the present study, Liangshan Semifine-wool, White Suffolk, and Butuo Black sheep had obvious genetic divergences, according to pairwise IBS distance. However, we could not differentiate Jialuo and Mage sheep because all the individuals were mostly clustered together. This is inconsistent with the former microsatellite marker-based report showing that Butuo Black and Jialuo sheep have a close genetic relationship [15]; this difference might arise from the different molecular markers used in the two studies. Overall, the three local pure breeds had closer genetic relationships with each other than with the composite breed of Liangshan Semifine-wool sheep and the exotic breed of White Suffolk sheep; this would indicate their respective genetic origins. The inter-breed F_ST_ values also supported this overall population structure for the five sheep breeds studied.

## Conclusion

In the present study, we successfully employed SLAF-seq and constructed a reference panel of genome-wide and high-quality SNPs in five sheep breeds in Sichuan, China. Their genetic diversity and population structures were subsequently revealed, which would be helpful to facilitate our understanding and conservation of these genetic resources.

## Supporting information

S1 Table Raw sequencing data in this study. (PDF)
