# Supplementary material for: Genetic diversity and population structure of sheep (Ovis aries) in Sichuan, China
Source: PLoS One. 2021 Sep 28;16(9):e0257974. doi: 10.1371/journal.pone.0257974 (PMC8478206; doi:10.1371/journal.pone.0257974)
Supplement: S1 Table — (PDF) [file pone.0257974.s001.pdf]

S1 Table. The raw data detected in this study

Sample ID	Total Reads	Total Bases	Q30 Percentage(%)	GC Percentage(%)
BT04	4,717,688	943,152,330	96.2	48.55
BT05	4,996,148	998,757,944	95.98	50.11
BT06	11,002,285	2,200,129,842	96.13	47.85
BT07	9,369,618	1,873,627,690	95.65	50.08
BT09	10,461,986	2,092,155,880	95.09	50.32
BT10	8,481,314	1,695,759,588	96.02	53.21
BT15	4,277,565	855,086,616	96.19	48.77
BT16	9,173,828	1,834,497,594	95.65	51.25
BT20	7,041,741	1,408,029,282	95.62	51.7
JL02	9,852,318	1,970,092,912	95.94	48.86
JL07	5,233,039	1,046,509,466	96.01	46.45
JL09	9,475,248	1,894,829,816	95.99	43.32
JL10	3,710,836	742,091,754	95.46	49.75
JL12	6,549,628	1,309,828,336	93.82	47.76
JL17	4,633,275	926,558,490	94.35	47.51
JL18	4,676,322	935,189,464	94.34	47.73
JL20	6,293,696	1,258,602,124	96.09	43.82
LS01	6,852,351	1,370,116,052	96.01	45.56
LS02	6,363,043	1,272,215,138	95.91	46.65
LS03	10,549,585	2,109,670,880	95.79	48.38
LS04	7,971,983	1,594,153,978	95.8	48.96
LS05	7,556,061	1,511,043,924	95.95	46.68
LS06	5,205,737	1,040,795,490	96.14	47.19
LS07	11,774,635	2,354,669,838	96	49.67
LS08	11,153,605	2,230,404,726	95.75	50.77
LS09	9,138,011	1,827,219,694	95.49	51.34
LS10	9,712,748	1,942,210,046	96.19	48.69
LS11	4,435,555	886,711,706	96.3	47.18
LS12	4,539,468	907,446,464	96	49.23
LS13	10,187,126	2,037,167,658	96.1	49.62
LS14	6,186,161	1,236,865,732	96.69	46.5
LS15	9,263,686	1,852,341,398	96.15	50.81
LS16	5,225,444	1,044,526,188	96.06	51.71
LS17	5,834,594	1,166,501,966	96.03	48.6
LS18	10,086,779	2,017,010,464	95.77	52.65
LS19	12,694,855	2,538,704,392	95.51	51.25

LT02	4,153,469	830,603,352	95.77	47.27
LT03	8,230,265	1,645,878,614	95.94	47.53
LT04	3,559,765	711,901,830	95.33	49.33
LT05	5,501,540	1,100,156,820	96.08	49.29
LT06	12,076,649	2,415,150,356	96.29	43.87
LT07	7,618,993	1,523,514,146	95.98	46.2
LT08	7,724,285	1,544,740,888	95.47	47.99
LT09	10,813,711	2,162,444,364	95.58	49.22
LT10	6,576,532	1,315,137,370	96.29	47.93
LT11	7,306,160	1,461,150,048	95.93	42.9
LT12	8,828,879	1,765,655,224	95.88	48.47
LT13	4,505,739	901,049,680	96.05	48.8
LT14	3,621,879	724,287,784	94.87	50.38
LT15	8,234,440	1,646,715,864	95.65	48.51
LT16	5,239,217	1,047,758,058	96.06	45.16
LT17	3,705,306	740,964,994	96.11	46.89
LT18	8,757,325	1,751,024,038	96.14	49.85
LT19	4,678,369	935,615,162	94.34	47.91
LT20	7,825,474	1,564,963,604	96.22	46.04
Total	506,545,353	101,293,913,012	96.01	48.23
WS01	5,708,107	1,141,529,302	95.1	49.01
WS02	5,468,869	1,093,575,412	96.17	48.34
WS03	3,007,528	601,458,034	94.58	46.99
WS04	38,835,332	7,766,917,736	92.62	44.05
WS06	3,957,257	791,380,884	95.95	49.22
WS09	5,733,866	1,146,702,068	93.88	46.71
WS11	4,980,096	995,956,826	94.34	46.39
WS12	4,030,825	806,100,362	94	48.98
WS13	4,733,332	946,616,880	93.25	47.76
WS14	3,226,546	645,245,676	94.84	48.46
WS16	4,446,112	889,147,908	94.24	46.97
WS17	4,368,788	873,664,128	93.97	49.98
WS18	7,004,451	1,400,707,776	96.28	43.33
WS20	7,408,285	1,481,522,962	96.04	49.45
